# ACE2/ACE imbalance mediates bisphenol A-induced lung injury in Wistar rats: Results from captopril versus losartan histo-biochemical study

**DOI:** 10.1016/j.heliyon.2023.e22056

**Published:** 2023-11-04

**Authors:** Ahmed A. Morsi, Ezat A. Mersal, Ahmed M. Abdelmoneim, Eman Mohamed Faruk, Mohamed M. Sofii, Nehad Ahmed Sadek, Khalid Elfaki Ibrahim, Hatem J. Aljanfawe, Iman Elmadhoun, Wejdan Mubarak, Mashael Malik Mahmoud, Mohamed S. Salim

**Affiliations:** a Department of Histology and Cell Biology, Faculty of Medicine, Fayoum University, Fayoum, Egypt; b Biochemistry Department, Faculty of Science, Assiut University, Assiut, Egypt; c Department of Basic Medical Sciences, Vision Colleges, Riyadh, Saudi Arabia; d Physiology Department, Faculty of Medicine, Fayoum University, Fayoum, Egypt; e Anatomy Department, College of Medicine, Umm Al-Qura University, Makkah, Saudi Arabia; f Department of Histology and Cytology, Faculty of Medicine, Benha University, Benha, Egypt; g Department of Anatomy and Embryology, Faculty of Medicine, Fayoum University, Fayoum, Egypt; h Department of Zoology, College of Science, King Saud University, P.O. Box 2455, Riyadh 11451, Saudi Arabia; i Vision Colleges, Riyadh, Saudi Arabia; j Medical Laboratory Technology Department, Higher Technological Institute of Applied Health Sciences, Beni-Suef, Egypt

**Keywords:** BPA lung injury, Inflammation/apoptosis, Altered renin-angiotensin system, Rat immunohistochemistry

## Abstract

Bisphenol-A (BPA) is a synthetic chemical compound broadly used in the plastic and epoxy resin industries with a considerable potential for food contamination. Literary reports have suggested that the altered renin-angiotensin system (RAS) is a mechanism for lung injury and inflammation caused by variable agents. The current study sought to investigate the contribution of RAS to BPA-induced lung damage. Moreover, the study assessed whether angiotensin II and/or bradykinin pathways were involved. For this aim, the angiotensin-converting enzyme (ACE) inhibitor captopril (Cap), either alone or combined with bradykinin receptor antagonist icatibant (Icat), was attempted versus the angiotensin receptor blocker losartan (Los). An eight-week study was conducted on forty Wistar male albino rats randomly divided into five equal groups: control, BPA, BPA/Cap, BPA/Los, and BPA/Cap/Icat groups. Captopril (100 mg/mL) and losartan (200 mg/mL) were given orally in drinking water, but icatibant (Icat) was injected subcutaneously (250 μg/kg) during the last two weeks of captopril treatment. Biochemical analysis of bronchoalveolar lavage fluid (BALF) and lung tissues, polymerase chain reaction (PCR) assay for *ACE*, *ACE2*, and *caspase-3* genes expression, and histological and immunohistochemical studies were carried out to evaluate BPA-mediated pulmonary inflammation/apoptosis. BPA impaired the histological structure of the lungs, increased ACE, ACE2, and caspase-3 expressions at both gene/protein levels, and increased BALF inflammatory cytokines and lung oxidative markers. Inhibiting the ACE activity by captopril maintained the histological lung injury score, restored inflammation and the ACE2/ACE balance, and decreased apoptosis. Further improvement was obtained by the angiotensin II receptor (ATR1) blocker losartan. Icatibant (bradykinin B2 receptor blocker) didn't counteract the observed captopril effects. It was strongly suggested that RAS contributed to BPA-induced lung damage via alteration of ACE2 and ACE expression mediating angiotensin II generation rather than bradykinin.

## 1 Introduction

Bisphenol A (BPA) is a well-known industrial chemical widely used in manufacturing epoxy resin and plastic resources and is associated with everyday human exposure via food contamination [1]. Regarding health hazards, BPA produces chronic inflammation, oxidative stress induction, DNA damage, and fibrotic reactions in many organs [2,3]. Several studies [4,5] demonstrated the toxic effect of BPA on the lungs and explored inflammation, oxidative stress, and apoptosis as common pathways for BPA-induced lung injury.

The renin-angiotensin system (RAS) is crucial for the pathogenesis of lung inflammation and respiratory distress [6]. Angiotensin converting enzyme (ACE) is an essential enzyme in both RAS and kallikrein-kinin system (KKS). In KKS, ACE breaks bradykinin into other metabolites, whereas it generates angiotensin II in the RAS [7]. Angiotensin II (Ang II) is an effective vasoconstrictor and leads to inflammation and apoptotic changes by activating the angiotensin II receptor type 1 (ATR1). Moreover, angiotensin II stimulates the release of IL-8 and IL-6, a well-known mediators in the inflammatory response of various origins. Besides, angiotensin II enables TNF-α facilitated necroptosis induced in the lung due to chemical toxicity [8,9].

Angiotensin-converting enzyme 2 (ACE2) is a structural homolog of ACE with a high sequence identity and similarity, and they are intensely expressed in lung tissues. ACE and ACE2 are membrane-bound zinc enzymes, but ACE2 forms angiotensin (1–9) from angiotensin I and generates angiotensin (1–7) from angiotensin II. Therefore, ACE2 has a dual role by both breaking down angiotensin II (vasoconstrictor) and producing the vasodilator angiotensin (1–7), so a negative feedback effect was exerted on the RAS [10]. Since ACE2 was initially demonstrated to be a functional entry receptor for the SARS coronavirus, the importance of RAS in lung injury and respiratory distress has been intensively emphasized and ACE2 has become a worthy area of research interest. The ACE/ACE2 system is composed of 2 balanced arms, the unfavorable adverse arm (ACE–Ang II–ATR1 receptor) and the protective counter-regulatory arm (ACE2–Ang 1-7–Mas receptor) [11].

Bradykinin is vital in inflammation initiating vascular leakage and cytokines induction [12]. It also showed anti-apoptotic and anti-fibrotic effects in a rat model of tacrolimus-induced kidney injury [13]. Therefore, ACE might facilitate lung injury via angiotensin II and or bradykinin pathways.

Literature studies [4,5] have proven BPA-induced lung injury; however, it is unknown whether BPA exposure could affect ACE and ACE2 expressions or their related signaling pathways. The current work investigated whether pulmonary ACE and ACE2 activities increased during BPA-induced lung toxicity in Wistar rats. Moreover, the study assessed whether angiotensin II and/or bradykinin pathways were involved. For this aim, the angiotensin-converting enzyme inhibitor captopril, either alone or combined with bradykinin receptor antagonist icatibant was attempted versus the angiotensin receptor blocker losartan to determine if the potential effects of ACE were achieved, whether via angiotensin II or bradykinin.

## 2 Materials and methods

### 2.1 Chemicals and drugs

Bisphenol-A was provided as white beads or granules with ≥99 % purity, catalog #: 239,658, Sigma-Aldrich, Saint Louis, MO, USA. It was dissolved in 30 % ethanol [14] and then diluted in normal saline to a concentration of less than 0.1 % ethanol [15]. Captopril (Capoten 50 mg tablets) was manufactured by Bristol Mayers Squibb (BMS), Egypt. Losartan 100 mg coated tablets were purchased from MEPACO pharmaceutical company, Cairo, Egypt. Icatibant, HOE-140 (Estereban 30 mg/3 mL pre-filled s.c. syringe) was purchased from Aroma pharmaceutical industry LTD, Ergene, Tekirdağ, Turkey. One ml of icatibant was diluted in 9 mL normal saline to a final concentration of 1 mg/mL.

### 2.2 Animals care

Forty adult male albino rats weighing about 190–210 gm were used. The animals were brought from VACSERA's breeding unit, the Holding Company for Biological Products and Vaccines, Giza, Egypt. For acclimatization, the animals had free access to rodent-specific food (rat standard chow diet, R04, Safe, France) and water without handling for one week. The rats were kept under appropriate living conditions (12-h light/dark cycle, at an ambient temperature of 25 ± 1 °C and 60 % humidity. To avoid the potential confounding sources of BPA, glass water bottles and polypropylene-made cages were used as housing precautions. The study was approved by the local ethics committee for scientific research, Faculty of Medicine, Fayoum University, Egypt (Approval #: R430/104-12Mars2023), which was consistent with the National Institutes of Health (NIH's) Guide for the Care and Use of Laboratory Animals [16]. All research settings regarding animal biosafety, animal handling, and reducing animal suffering, distress, and pain were followed.

### 2.3 Animals’ groups and treatment

The animals were randomly assigned into five groups, eight rats each:

**Control group:** The rats were allowed free access to distilled water (drinking water) during the study. They were given the same volume of saline containing 0.1 % ethanol orally (vehicle for BPA) for eight weeks.

**BPA-exposed group**: The rats received BPA (10 μg/kg/d) via oral gavage for eight weeks.

**BPA/captopril-treated (BPA/Cap) group**: The animals received captopril in drinking water 100 mg/L [17] and BPA (10 μg/kg/d) via oral gavage for eight weeks.

**BPA/losartan-treated (PBA/Los) group**: The animals received losartan in drinking water 200 mg/L [18] and BPA (10 μg/kg/d) via oral gavage for eight weeks.

**BPA/captopril/Icatibant-treated (BPA/Cap/Icat) group:** The animals received captopril in drinking water 100 mg/L [17], BPA (10 μg/kg/d) via oral gavage for eight weeks. After six weeks, five rats received a daily single subcutaneous (s.c.) injection of icatibant 250 μg/kg [19] for the last two weeks. The remaining three rats acted as control animals and received s.c. saline injection (instead of icatibant) for the same two weeks.

In this study, low-dose chronic BPA exposure was chosen as it seems to be relevant to public health exposure. Acute human toxicity due to massive BPA exposure is unexpected in the real world. The utilized dose of BPA was 2.5 folds the Tolerable Daily Intake (TDI, 4 μg/kg/day) adopted by the European Food Safety and Authority (EFSA panel) [20].

### 2.4 Bronchoalveolar lavage fluid (BALF) collection

For euthanasia of rats, all animals were anesthetized 24 h after the last dosing, using intramuscular ketamine/xylazine (80/10 mg/kg) injection, according to Tsukamoto et al. [21] then humanely killed via cervical dislocation. The BALF samples were collected from three animals per group; those animals were not used for histological and immunohistochemical examinations. In each rat, the chest was incised, thoracic organs were dissected, and the trachea was cannulated. Lung wash was conducted thrice through the endotracheal cannula using 2 mL phosphate buffer saline (PBS) with a gentle massage. Individual BALF samples were pooled with ∼80 % recovery volume. Centrifugation (4000 rpm for 5 min) was done for each specimen. The cell pellets were used for microscopy-based cell counting of the total leukocytes via a hemocytometer. Per slide, at least 200 cells were counted at x400 magnification. The BALF's supernatants were analyzed for interleukin-6 (IL-6), tumor necrosis factor-alpha (TNF-α), transforming growth factor-beta 1 (TGF-β1), Ang II, and bradykinin using rat-specific ELISA kits according to the guidelines provided by the manufacturers (Elabscience Biotechnology Inc, USA). The ELISA sensitivity was 7.5 pg/mL for IL-6, 9.38 pg/mL for both TNF-α and Ang II, 0.1 ng/mL for TGF-β1, and 0.75 ng/mL for bradykinin.

### 2.5 Lung tissue collections

In a separate set of animals (n = 5), the lungs were removed without lavage. For each rat, the left lung was refrigerated at −20 °C for a PCR study and for tissue homogenate measurements of malondialdehyde (MDA), superoxide dismutase (SOD), TGF-β1, and Ang II. The lung tissues were homogenized in 10 mL cold buffer at pH 7.5. Centrifugation (4000 rpm, 10 min) was done, and the supernatant was collected for analysis. Thiobarbituric acid reactive substances (TBARS) assay has been utilized as an indicator for MDA measurement [22]. SOD was measured as mmoL/min/mg protein according to the method adopted by Sun et al. [23]. Angiotensin II was analyzed by ELISA following the manufacturer's instructions. The right lungs were fixed in formol saline in preparation for paraffin microtechniques.

### 2.6 ACE, ACE2, and caspase-3 mRNA expression quantification

The lung tissues assigned for PCR were homogenized entirely. cDNA was initially synthesized from the mRNA using a PCR Master Mix kit (catalog #: 4440040, Applied Biosystems). The PCR reaction was evaluated by StepOneSystem (RQ Manager 1.2, software v 2.1, Applied Biosystem, USA). The targeted genes (ACE, ACE2, and caspase-3) were normalized to *GAPDH* (an endogenous housekeeping gene) using the comparative 2^–ΔΔCt^ method for the determination of the relative fold gene expression, as reported by Livak and Schmittgen [24]. For cDNA synthesis, the thermocycler was set at 45 °C for 15 min, followed by 95 °C, for 5 min, for polymerase activation. For detection of the rat *ACE*, *ACE2*, and *caspase-3* transcripts, the primer sequences were shown in Table 1. They were obtained from NCBI's GenBank (http://www.ncbi.nlm.nih.gov/tools/primer-blast).

**Table 1:** Sequences and accession numbers of the used PCR primers.

Gene	Accession number	Primer sequence (5′ → 3′)
Cysteine aspartic acid-specific protease-3 *(Caspase-3)*	NM_012922.2	F: GAGCTTGGAACGCGAAGAAA R: TAACCGGGTGCGGTAGAGTA
Angiotensin-converting enzyme *(ACE)*	NM_012544.1	F: CTACTGGCGCTCCTGGTATG R: ACGTGCAGGTCCTTGTACTG
Angiotensin-converting enzyme-2 *(ACE2)*	NM_001012006.2	F: GAATGCGACCATCAAGCGTC R: GGCTCAGTCAGCATGGAGTT
Glyceraldehyde 3- phosphate dehydrogenase (*GAPDH*)	NM_017008.4	F: GCCAGCCTCGTCTCATAGAC R: AGTGATGGCATGGACTGTGG

Notes: F: forward, R: reverse.

### 2.7 Histology and immunohistochemistry

The lung samples assigned for histology and immunohistochemistry (IHC) were fixed, for two days, in formol saline (10 %, at room temperature) for paraffin microtechnique preparation. The tissue samples underwent dehydration by serial immersion in ethanol concentration grades (70 %, 90 %, and 100 %), cleared (in xylene), and infiltrated and embedded (in paraffin). For microtomy, 5-μm cut sections were obtained and prepared for histological stains. The deparaffinized sections were rehydrated in descending ethanol grades and exposed to Hematoxylin & Eosin (H&E) and Masson trichrome staining [25]. For IHC, positively charged glass slides were used to mount the formalin-fixed paraffin-prepared lung sections. The mounted slides were subjected to the in-sequence steps of the IHC technique [26] to identify ACE, ACE2, and caspase-3 proteins. The sections were deparaffinized, rehydrated, and boiled in citrate buffer for antigen retrieval (10 min, pH 6). The slides were left for natural cooling, then subjected to 10-min incubation in H_2_O_2_ for blockage of the endogenous peroxidase activity. The next step was incubation with ACE (1:300, Cat #: E-AB-70116), ACE2 (1:300, Cat #: E-AB-12224) and caspase-3 (1:200, Cat #: E-AB-63602) primary antibodies for 24 h at room temperature. They are all rabbit-prepared polyclonal antibodies purchased from Elabscience Biotechnology Inc, USA. Tris buffer-based antibody dilutant was used for the dilution of the primary antibodies. Anti-polyvalent HRP/DAB rabbit-specific detection system (TP-015-HD, Lab Vision™, Thermo Fisher Scientific) was utilized to visualize and complete the reaction. Hematoxylin was used as a nuclear counterstaining. The immunoreaction was identified by cytoplasmic immunopositivity. The positive controls were rat testis, human lung cancer, and human breast cancer tissues for ACE, ACE2, and caspase-3 respectively. Negative control slides were prepared by canceling the primary antibody incubation. Slide examination, photography, and morphometric measurements were conducted in the Digital Pathology Lab, Pathology Department, Faculty of Veterinary Medicine, Cairo University, Egypt. Leica DM4 B upright microscope provided with a built-in Leica DMC 4500 digital camera (Leica, Microsystems, Wetzlar, Germany) was used.

### 2.8 Morphometric analysis

Blind morphometric analysis was performed by two experts in the field. Leica Application Suite X software (LAS X, Leica Microsystems, Wetzlar, Germany) was used for analyzing the results and was used for measurements. Trichrome-stained slides were used to quantitatively evaluate fibrosis using the area percent of collagen fiber distribution at x200 magnification. The H & E-staining was used to assess the lung injury score (LIS). For measurement of LIS, four parameters (cell infiltration, hyaline membrane formation, edema, and septal thickening) were assessed and scored in a minimum of 20 random fields (at x400 total microscopy magnification) according to the lung injury scoring system published by Kadam et al. [27]. The alveolar and bronchiolar wall thicknesses were also evaluated. The ACE, ACE2, and caspase-3 protein immunoexpressions were quantitatively assessed. For such purpose, the area percent of ACE and ACE2 and the optical density of caspase-3 expression were measured in ten non-overlapping random fields at x200 magnification.

### 2.9 Statistical analysis

The research data were subjected to statistical analysis using Windows-installed GraphPad prism v8.0 software (GraphPad Software Inc., San Diego, CA, USA). Initial data preparation and normality check were done by Kolmogorov–Smirnov test. All data were shown as a mean ± standard deviation (SD). Statistical significance between the study groups was detected using One-way Analysis of Variance (ANOVA). Tukey's post hoc test was applied for the detection of group differences. Statistical significance was applied at *p* < 0.05.

## 3 Results

### 3.1 Deaths and clinical status of the animals

No premature death of the rats was recorded before eight weeks. No respiratory distress, irritability, agitation, or other behavioral changes were noticed during the study.

### 3.2 Total leucocytic count in BALF

As a marker of inflammation and lung injury, the total leukocytes were counted in the individual BALF samples (Table 2). Compared to the control total BALF leukocytes, BPA-alone exposed rats showed a marked increase (p < 0.001), which was reversed by captopril (p = 0.007 vs. BPA), losartan (p < 0.001 vs. BPA), and Cap/Icat combination (p < 0.001 vs BPA). Losartan treatment showed a dampening effect on leucocytes count (p = 0.002 vs Cap). Icatibant combined with captopril pretreatment showed no significant change compared to captopril-alone treatment.

**Table 2:** Effect of captopril, losartan, and BK receptor antagonist icatibant on the BALF's total leukocytes, bradykinin, Ang II, and inflammatory mediators' levels in BPA-induced lung injury at the end of the experiment.

	Total leucocyte count (x 10^5^)	IL-6 (pg/ml)	TNF-α (pg/ml)	TGF-β (pg/ml)	Angiotensin II (pg/ml)	Bradykinin (pg/ml)
Control	1.1 ± 0.3	27.99 ± 2.5	31.75 ± 3.2	13.03 ± 2.8	91.77 ± 5.2	0.141 ± 0.04
BPA	7.5 ± 0.5^a^	175.5 ± 4.8^a^	116.2 ± 4.1^a^	56.43 ± 3.7^a^	227.3 ± 20.8^a^	0.683 ± 0.01^a^
BPA/Cap	6 ± 0.1 ^b^	47.9 ± 2.4 ^b^	44.19 ± 3.3 ^b^	20.4 ± 3 ^b^	127.7 ± 2.6 ^b^	0.101 ± 0.01 ^b^
BPA/Los	3.7 ± 0.3 ^bc^	37.8 ± 2.5 ^bc^	32.96 ± 3.2 ^bc^	12.3 ± 3.5 ^bc^	155.3 ± 4.3 ^bc^	0.112 ± 0.01 ^bc^
BPA/Cap/Icat	5.6 ± 0.6 ^b^	49.12 ± 2.2 ^b^	44.44 ± 5.6 ^b^	19.3 ± 1 ^b^	125.9 ± 3.4 ^b^	0.152 ± 0.05 ^b^

The data are shown as a mean ± SD (n = 3). One-way analysis of variance, followed by multigroup comparison Tukey's test, was used at *p* < 0.05. ^a^: versus control, ^b^: versus BPA group, and ^c^: versus BPA/Cap group.

### 3.3 Bradykinin, angiotensin II, and inflammatory mediators’ levels in BALF

Eight-week BPA exposure led to a marked rise in the BALF levels of IL-6, TNF-α, TGF-β, Ang II, and bradykinin compared to the control levels (Table 2). They all increased by ∼5, ∼2.5, ∼3, ∼1.5, and ∼ 4-folds, respectively (p < 0.001 versus control). Captopril treatment didn't increase BK levels. Losartan was superior to captopril (p < 0.001) in maintaining the measured parameters (i.e. preventing the BPA-induced biochemical changes). Captopril was effective in preserving Ang II level (p < 0.001 versus losartan). Icatibant injection combined with captopril didn't significantly change the levels of IL-6, TNF- α, and TGF-β compared to the captopril alone-treated group. Bradykinin slightly increased; however, this increase wasn't statistically significant.

### 3.4 Lung angiotensin II concentrations and oxidative stress status

**Fig. 1:**
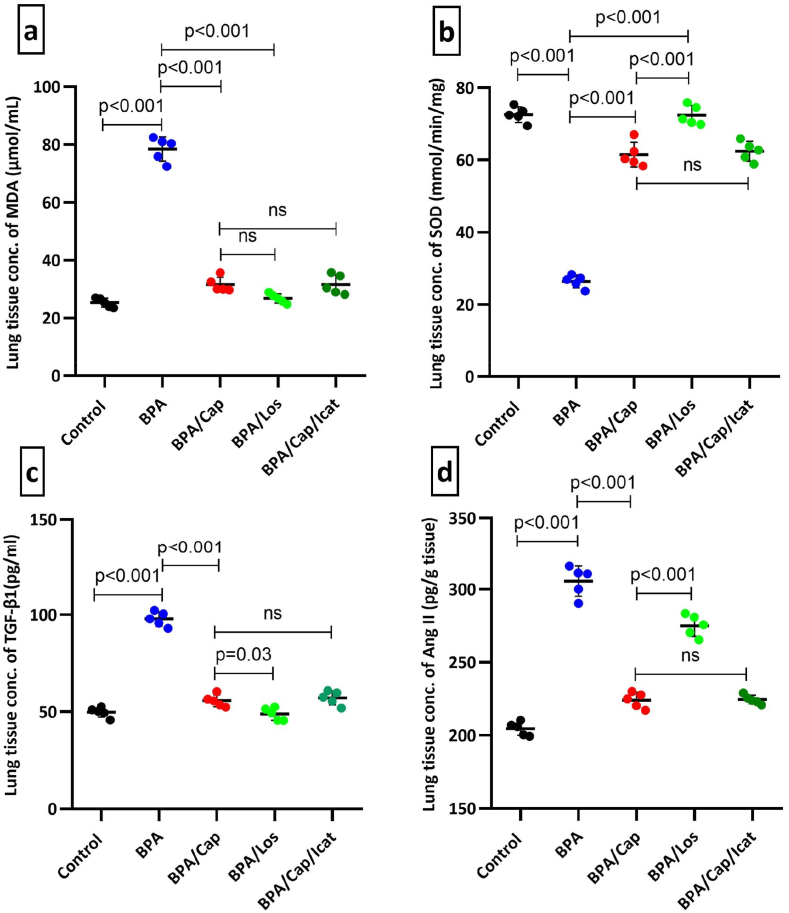
Scatter plot graph of the lung tissue concentrations of MDA (a), SOD (b), TGB-β1 (c), and angiotensin II (d) in the individual lung samples of BPA-exposed rats treated by captopril, losartan, and BK receptor antagonist icatibant at the end of the experiment. The data are shown as a mean ± SD (n = 5). One-way Analysis of Variance, followed by multigroup comparison Tukey's test was used at *p* < 0.05. Statistical significance between the groups and the p-value are shown in the graph.

The lung tissue homogenates showed a remarkable increase in the MDA, TGF-β1, and Ang II concentrations in the BPA-exposed group (Fig. 1). MDA, TGF-β1, and Ang II (Fig. 1a, c, and d, respectively) showed an increase by 2, ∼1, and ∼0.5 folds, respectively (p < 0.001 vs control). Captopril and losartan maintained the measured parameters with more improvement noted in the losartan-treated group (p < 0.05 vs captopril). Although losartan treatment decreased MDA levels more than captopril treatment, it was statistically nonsignificant. Captopril was effective in maintaining Ang II tissue level (p < 0.001 versus losartan). Cap/Icatibant combination didn't produce major changes in both variables compared to captopril alone treatment. On the other hand, the SOD activity (Fig. 1b) was decreased by 0.64-fold in the BPA-exposed group (p < 0.001 vs. control) and significantly elevated due to either captopril or losartan treatment (p < 0.001 vs BPA group). Icatibant injection combined with captopril (Cap/Icat group) didn't affect SOD compared to captopril alone group.

### 3.5 PCR analysis of ACE, ACE2, and caspase-3 mRNA expression in lung tissues

**Fig. 2:**
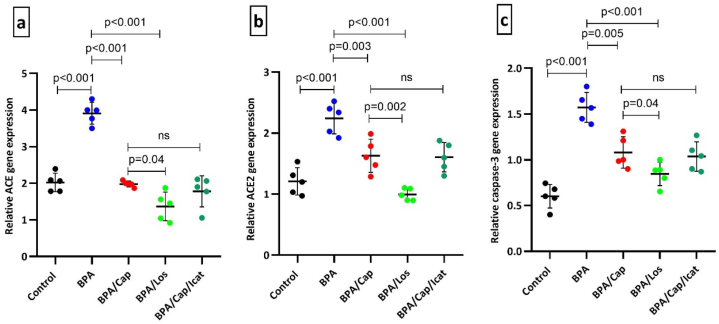
Scatter plot graph of the individual ACE (a), ACE2 (b), and caspase-3 (c) gene expression measured by PCR in the lung samples of BPA-exposed rats treated with captopril, losartan, and icatibant (n = 5). The data are displayed as mean ± SD. Statistical significance between groups is indicated by the horizontal lines. One-way ANOVA, followed by post hoc Tukey's test, was used, and the *p*-value is shown in the graphs.

PCR examination of the lung samples revealed major changes in the ACE, ACE2, and caspase-3 gene expressions (Fig. 2). The ACE gene expression increased by 0.93-fold (p < 0.001 vs. control) in the BPA-exposed rats; meanwhile, the ACE2 showed a 0.85-fold increase (p < 0.001 vs control), indicating a reduced ACE2/ACE ratio. The apoptotic gene caspase-3 was also significantly upregulated in the BPA-exposed group (1.6-fold increase, p < 0.001 vs control). Captopril and losartan treatment significantly maintained the expression of the three genes (p < 0.05 vs BPA). Losartan had more preventive effect than captopril (p < 0.05). Icatibant injection combined with captopril treatment did not affect the genes’ expression compared to the captopril alone treatment.

### 3.6 Lung histology and lung injury score

**Fig. 3:**
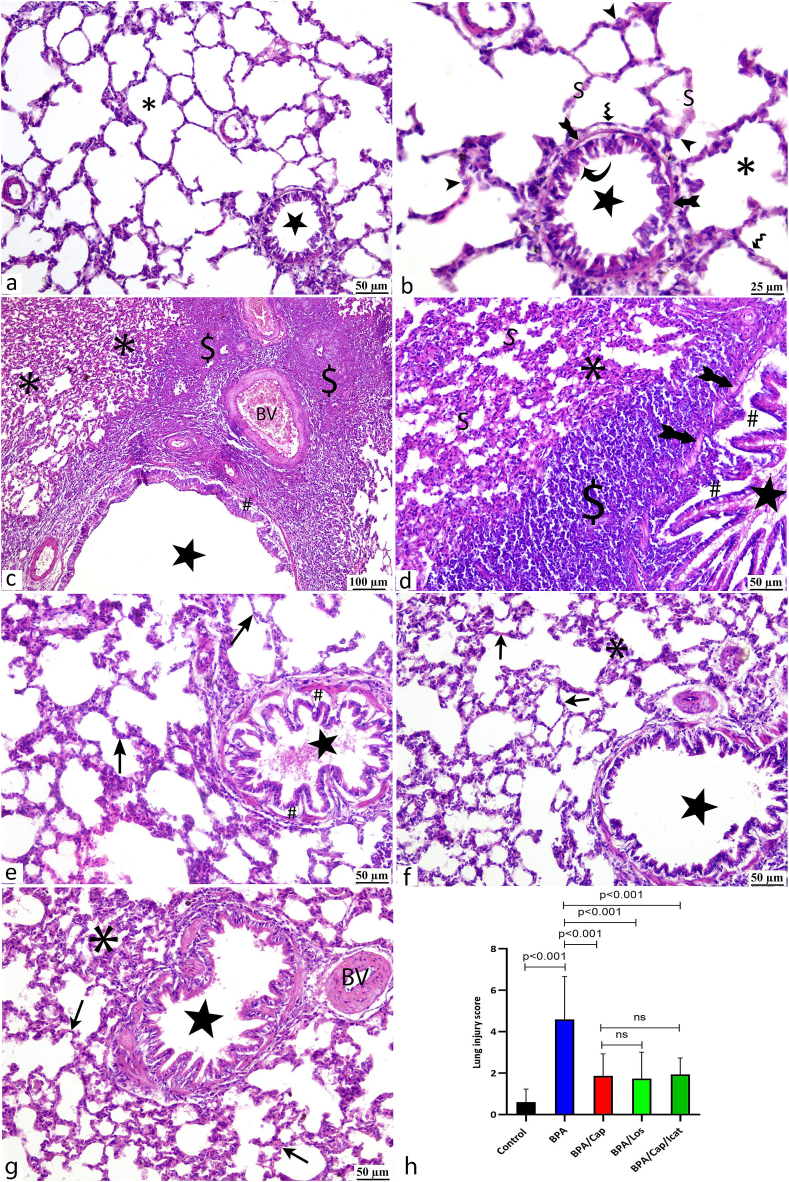
H&E-stained lung sections of the different experimental groups at the end of the study showing the histological structure of lung bronchioles (stars) and alveoli (*). Control group (a, b) shows the typical microscopic structure of normal lung tissues. The alveoli (*) have thin septa (S) and are lined by flat type I pneumocytes (zigzag arrows) and cubical type II pneumocytes (arrowheads). The bronchioles (star) have a thin smooth muscle layer (arrows with tails) and are lined by columnar cells (curved arrows). BPA-alone exposed group (c, d) shows massive peribronchiolar (star) and perivascular (BV) mononuclear inflammatory cell infiltrations ($). The bronchiolar wall shows thick smooth muscle layer (arrows with tails) and subepithelial wide spaces (#) indicating edema. The alveoli (*) show narrowed air spaces. The vascular walls (BV) and interalveolar septa (S) are thickened. BPA/Cap (e), BPA/Los (f), and BPA/Cap/Icat (g) groups show seemingly healthy lung parenchyma. The lung alveoli (*) have thin walls (arrows). The bronchioles (stars) appear normal except for a few subepithelial edema (#) in BPA/Cap group. Bar graph (h) shows quantitative evaluation of the lung injury score displaying data as a mean ± standard deviation (n = 5). Statistical significance between groups and the *p*-value are shown in the graph. One-Way ANOVA and post hoc Tukey's tests were used at *p*-value <0.05. Scale bar. b: 25 μm; a, *d*–g: 50 μm; c: 100 μm.

Microscopic examination of H&E-stained control lung tissues showed the typical histological structure of lung parenchyma. The bronchioles were lined by columnar epithelial cells and had average wall thickness. Intact alveoli appeared inflated and thin-walled and were lined by two different cell types: flat-shaped type I and cubical type II pneumocytes (Fig. 3a and b). BPA-alone exposed rats showed apparent histopathological alterations. The lung sections exhibited diffuse peribronchiolar and perivascular cellular infiltration, marked thickening of the interalveolar septa due to septal edema and cell infiltrations, narrow alveolar spaces, and multifocal fibrotic lesions. The bronchiolar wall showed edema indicated by subepithelial separations (Fig. 3c and d). All treated (BPA/Cap, BPA/Los, and BPA/Cap/Icat) groups showed almost normal pulmonary tissues in all the examined sections (Fig. 3e, f, g respectively).

**Fig. 4:**
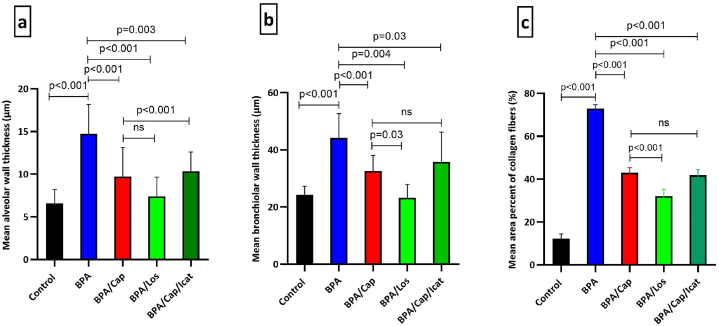
Quantitative evaluation of the alveolar wall (a), bronchiolar wall (b) thicknesses in the H &E-stained sections, and lung fibrosis (c) identified by Masson staining. The findings are displayed as a mean ± standard deviation (n = 5). Statistical significance between groups and the *p*-value are shown in the graphs. One-Way ANOVA and post hoc Tukey's tests were used at *p*-value <0.05.

For quantitative evaluation of the histological results, the lung injury score (LIS) was calculated (Fig. 3h), and the alveolar and bronchiolar wall thicknesses were measured (Fig. 4a and b respectively). The scoring system indicated an elevated LIS in the BPA-alone exposed animals (p < 0.001 vs control). All treated groups showed a decreased LIS (p < 0.001 vs BPA-alone group). No significant difference was noticed when comparing captopril alone versus captopril/icatibant combination or captopril alone versus losartan therapy. Statistical analysis of the alveolar and bronchiolar measurements exhibited thickened alveolar and bronchiolar walls in the BPA-alone group (p < 0.001 vs. control). All treatment groups showed maintenance of the alveolar and bronchiolar wall thickness (p < 0.05 vs BPA).

### 3.7 Lung fibrosis and collagen deposition

**Fig. 5:**
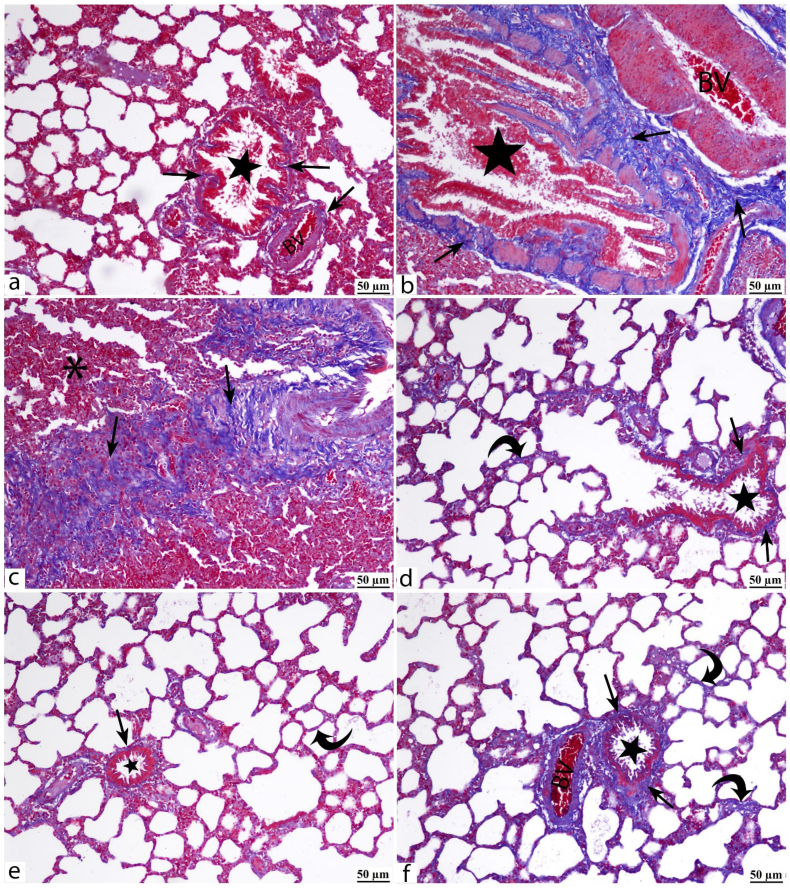
Fibrotic changes of the lung tissues of the different study groups is demonstrated by Masson trichrome-stained blue collagen fibers (arrows). Light microscopic examination of the control group (a) shows scarce fine collagen fibers around bronchioles (star) and blood capillaries (BV). BPA-alone group (b, c) shows diffuse peribronchiolar (*) and perivascular (BV) deposition of coarse collagen fibers. Also, fibrotic lesions (arrows) are seen interstitial among the obliterated alveoli (star). Cap (d), Los (e), and Cap/Icat (f) treatment groups show minimal collagen fibers around bronchioles (stars), blood vessels (BV), and within the alveolar septa (curved arrows). Scale bar: 50 μm.

Microscopic examination of the Masson trichrome-stained control lung sections showed normal distribution of delicate, blue-stained collagen fibers (Fig. 5a). The BPA-alone exposed group showed extensive fibrosis marked by a peribronchial, perivascular, and parenchymal (interstitial) accumulation of coarse collagen fibers (Fig. 5b and c). All treated groups showed a noticeable decrease in the collagen fibers (Fig. 5d, e, f). Quantitative evaluation of the fibrotic changes (Fig. 4c) showed a great mean area percent in the BPA-exposed rats (p < 0.001 vs control). Losartan treatment had a better effect than captopril (p < 0.001). Icatibant injection combined with captopril pretreatment had no significant impact compared to captopril-alone treatment.

### 3.8 ACE and ACE2 immunoreactivity in lung tissues

**Fig. 6:**
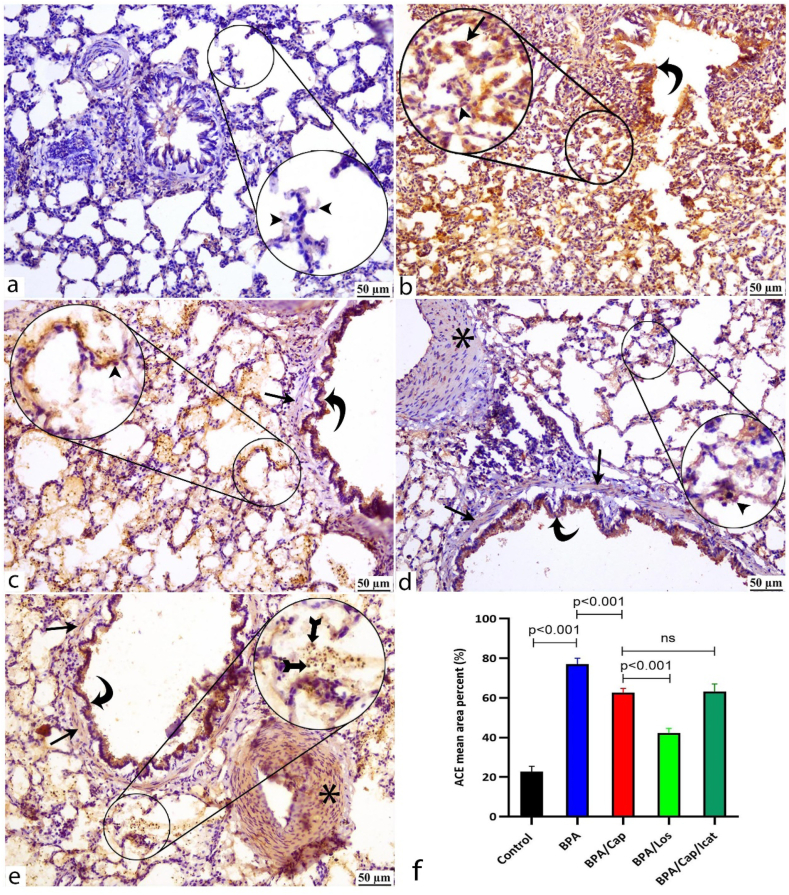
ACE protein expression is detected by immunohistochemistry (IHC) in the lung tissues of the study groups. Zoom insets are introduced in the figures for magnification of certain histological structures in the field. Control group (a) shows minimal ACE immunoexpression mainly stains the alveolar pneumocytes (arrowheads). BPA-alone group (b) shows strong diffuse parenchymatous ACE immunoexpression staining the bronchiolar epithelium (curved arrow), the thickened alveolar walls, and the lining type I (arrowhead), type II (arrow) pneumocytes. BPA/Cap (c), BPA/Los (d), and BPA/Cap/Icat (e) groups show an obvious decrease in the ACE expression with strong intensity elicited in the bronchiolar epithelium (curved arrow), alveolar pneumocytes (arrowheads). Mild to moderate vascular (asterisk) and bronchiolar (arrows) smooth muscle immunoreactivity is observed. BPA/Los group (d) shows a notable decrease in the ACE area of immunopositivity. Bar graph (f) shows the quantitative measurements of the area percent of ACE immunoreactivity. The data are shown as mean ± SD, n = 5. The *p*-value is shown, and statistical comparisons between groups are indicated by horizontal lines using post hoc Tukey's test. ns: nonsignificant. Scale bar: 50 μm.

**Fig. 7:**
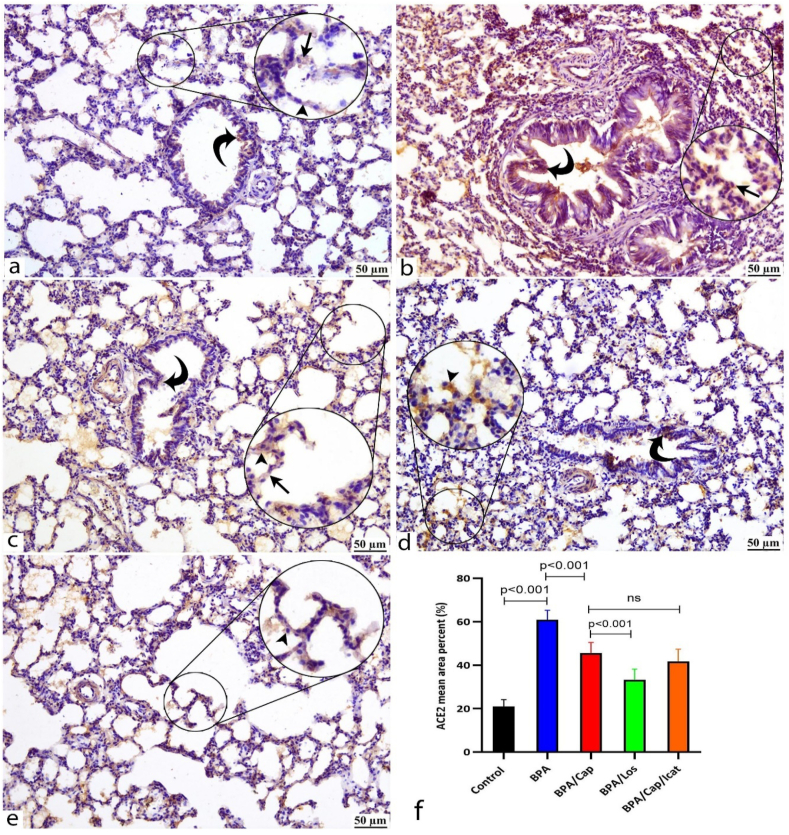
ACE2 protein expression is detected by immunohistochemistry (IHC) in the lung tissues of the study groups. Zoom insets are introduced in the figures for magnification of certain histological structures in the field. Control group (a) shows minimal ACE2 expression mainly stains the alveolar pneumocytes type I (arrowhead) and II (arrow) and, to some extent, the bronchiolar epithelium (curved arrow). BPA-alone group (b) shows moderate diffuse ACE2 immunoexpression staining the thickened alveolar septa, the lining alveolar pneumocytes (arrow), and partially expressed in the bronchiolar epithelium (curved arrow). BPA/Cap (c), BPA/Los (d), and BPA/Cap/Icat (e) groups show obvious decrease in the ACE2 expression limited to the alveolar type I (arrows) and type II (arrowheads). The bronchiolar epithelia (curved arrows) show very minimal ACE2 immunoreactivity. Bar graph (f) shows the area percent of ACE2 immunoreactivity. The data are shown as mean ± SD, n = 5. The *p*-value is shown, and statistical comparisons between groups are indicated by horizontal lines using post hoc Tukey's test. ns: nonsignificant. Scale bar: 50 μm.

ACE and ACE2 are generally expressed in the control lung tissue (Fig. 6, Fig. 7a, respectively). BPA enhanced their pulmonary expression with diffuse immunoreactivity staining the lung parenchyma (Fig. 6, Fig. 7b). Captopril and losartan treatments regulated the pulmonary expression of ACE (Fig. 6c and d respectively) and ACE2 (Fig. 7c and d respectively) with an outstanding effect in the losartan group (Fig. 6, Fig. 7d). Icatibant treatment didn't lead to noticeable changes (Fig. 6, Fig. 7e) compared to the captopril-alone treatment (Fig. 6, Fig. 7c). Quantitative measurements revealed a significant increase in the mean area percent of ACE and ACE2 expressions (Fig. 6, Fig. 7f respectively) in the BPA-exposed group (*p* < 0.001 respectively vs control). A substantial ACE2/ACE ratio imbalance was induced by BPA exposure. The mean area percent of ACE immunoreactivity increased by 2.4 folds (*p* < 0.001 vs. control) and was coupled with a 1.9-fold increase in ACE2 area percent (*p* < 0.001 vs control). As a result of the inconsistent increase, the ACE2/ACE ratio was reduced and disequilibrium of the two arms of the renin-angiotensin system (RAS) occurred. Losartan had a greater impact than captopril in maintaining (*p* < 0.001 vs. Cap group) the ACE and ACE2 expressions. In BPA/Cap/Icat group, icatibant had no significant effect on the expression of both proteins compared with the captopril-alone treatment (BPA/Cap group).

### 3.9 Lung apoptosis and caspase-3 immunoexpression

**Fig. 8:**
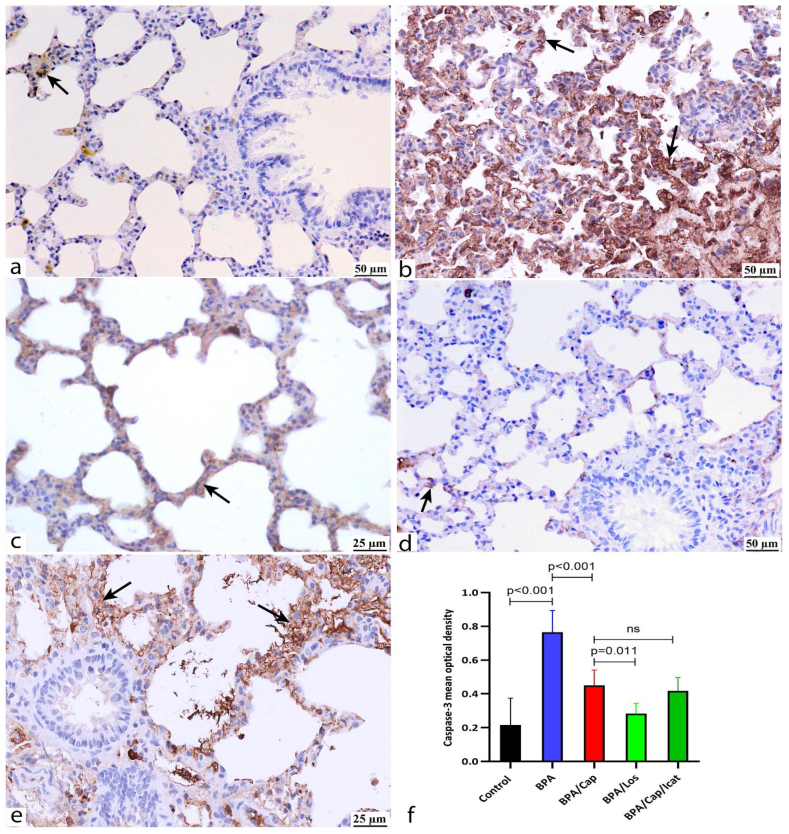
Caspase-3 protein immunoexpression in the lung tissues of the study groups at the end of the experiment. The positive immunohistochemical (IHC) reaction is observed as brown cytoplasmic coloration marked by arrows. The IHC staining shows almost negative caspase-3 expression with scarce caspase 3-stained cells in the control group (a). BPA-exposed group (b) shows extensive diffuse caspase-3 immunoexpression staining alveolar epithelium and alveolar septa. BPA/Cap and BPA/Cap/icat groups (c and e, respectively) show less intense caspase-3 positive cells. BPA/Los group (d) shows diffuse negative reaction with scarce caspase-3 positive cells. Bar graph (e) shows the mean optical density of caspase-3 immunopositivity at the end of the experiment. The data are shown as mean ± SD, n = 5. The *p*-value is shown, and statistical comparisons between groups are indicated by horizontal lines using post hoc Tukey's test. ns: nonsignificant. Scale bars: a, b, d: 50 μm; c, e: 25 μm.

The caspase-3 immunopositive cells were taken as an indicator of apoptotic changes (Fig. 8). BPA-exposed rats showed a marked caspase-3 immunoreactivity staining the alveolar pneumocytes and alveolar septa (Fig. 8b). Captopril (Fig. 8c), losartan (Fig. 8d), and Cap/Icat (Fig. 8e) combination decreased the caspase-3 immunoexpression. Quantitative comparison (Fig. 8f) showed a high mean optical density of caspase-3 immunoexpression in the BPA-exposed animals (p < 0.001 vs control). All treatments significantly decreased the caspase-3 immunoreactivity (p < 0.001 vs BPA group). Losartan showed a better improvement (p = 0.011 vs. BPA/cap) but not in icatibant treatment (p = 0.96 vs. BPA/cap).

### 3.10 Contribution of angiotensin II and or bradykinin to BPA-induced lung injury

Captopril treatment suppressed inflammation and the apoptotic marker caspase-3 in the BPA-exposed group. Specific receptor blockers were used to evaluate the involvement of either angiotensin II or bradykinin in the histological changes, inflammation, and apoptosis observed in the BPA group. If the effects of ACE on such changes were facilitated by Ang II, blocking its receptor (ATR1) could produce a similar effect as blocking ACE with the ACE inhibitor captopril. Indeed, Ang II receptors blocking by losartan treatment led to similar or even decreased BALF cytokine levels, inflammation (Table 2), oxidative stress (Fig. 1), and apoptosis (Fig. 8) as observed after captopril treatment. On the opposite, if the observed effects of ACE inhibitors were facilitated by the increased bradykinin concentrations, antagonizing its receptor by icatibant after captopril pre-treatment might restore the inflammatory and apoptotic changes. However, Bradykinin receptor blocker icatibant (HOE-140) injection after captopril pre-treatment showed no statistical difference compared to captopril-alone treatment (Fig. 1, Fig. 3, Fig. 5, Fig. 6, Fig. 7, Fig. 8).

## 4 Discussion

Lung inflammation, fibrosis, and apoptosis were the main findings in the current BPA-induced lung injury rat model. The entangling of the ACE2/ACE system as a putative mechanism underlying the pulmonary histological and biochemical changes induced by BPA has not been addressed. The present study reported a loss of the balanced ACE2/ACE system and a reduction in the ACE2/ACE ratio. The findings revealed 0.93- and 0.85-fold increases in ACE and ACE2 gene expression, respectively; meanwhile, a 2.4-fold-increase in ACE protein expression was noticed versus a 1.9-fold-increase for ACE2. Blockage of the renin-angiotensin system by angiotensin-converting enzyme inhibitor captopril or by the angiotensin II receptor (ATR1) blocker losartan reversed the histological and biochemical pulmonary disturbances induced by BPA exposure. Two-week-icatibant injection combined with captopril pre-treatment didn't significantly differ compared to captopril-alone treatment.

Although the airway inflammatory cell infiltration and the rapid release of chemical mediators were postulated mechanisms for lung injury induced by chemicals and toxicants [28], there was a lack of data supporting the ongoing processes underlying the progressive pulmonary damage. The renin-angiotensin system has been identified to maintain cardiovascular homeostasis and control blood pressure. Meanwhile, the activation of lung RAS and the disequilibrium between its pro/anti-inflammatory arms might precipitate inflammation, hence the pathogenesis of lung damage and respiratory distress [29,30].

Bisphenol-A exposure significantly increased ACE and ACE2 at gene/protein levels, as identified by PCR and immunohistochemistry. So, the involvement of pulmonary RAS in such lung injury process was suggested. The unbalanced increase of both proteins resulted in ACE2/ACE disequilibrium and reduced ACE2/ACE ratio with a shift toward the unfavorable arm of the RAS system (ACE/Ang II/ATR1). Indeed, the histological sections showed parenchymatous lung damage with an elevated lung injury score, inflammation, fibrosis, and caspase 3-mediated apoptotic changes. Also, the biochemical results of lung tissue and BALF analyses showed a high angiotensin II level and confirmed a state of inflammation and oxidative stress.

Similarly, Mansour et al. [3] and Rehman et al. [31] reported rat lung inflammation and oxidative stress induced by BPA. In the current BPA-induced lung injury, the authors proposed dominance of the harmful component of the RAS system, ACE expression upregulation, relative decrease of ACE2 expression, and angiotensin II upregulation. This suggestion was supported by the biochemical findings of BALF and lung tissue analysis, which revealed an elevated angiotensin II level. The authors' assumption was supported by Li et al. [32], who found ACE/ACE2 imbalance in both in vitro and in vivo models of lipopolysaccharide-induced lung injury in rats. They recorded an increase in ACE and a decrease in ACE2 expression. In a different lung injury model induced by ischemia-reperfusion (IR), Wang et al. [33] recorded an impaired activity of the protective ACE2 enzyme in the IR lung tissues and was reversed by the ACE2 activator diminazen aceturate. In the authors’ opinion, the increased ACE2 expression might be a compensatory mechanism attempted by the body to counteract the ACE adverse effects mediated by the ACE/angiotensin II axis.

Yilin et al. [34] identified two hemodynamic and non-hemodynamic mechanisms for lung injury induced by Ang II. Pulmonary vasoconstriction, pulmonary hypertension, and increased pulmonary capillary permeability are all part of the angiotensin II hemodynamic role. Meanwhile, the non-hemodynamic pro-inflammatory mechanism includes inflammatory cellular infiltration, TGF-β1 release, and lung fibrosis leading to augmented lung damage. Our research findings were consistent with the non-hemodynamic mechanism proposed by Yilin et al., 2015.

The BPA-induced lung damage was maintained by both captopril (BPA/Cap group) and losartan (BPA/Los group) treatments which showed an equal amelioration of the lung injury score in the H & E findings. However, losartan treatment was superior in restoring the biochemical alterations and the lung expression of ACE, ACE2, and caspase 3. In BPA/Cap/Icat group, the bradykinin receptor antagonist (icatibant) didn't counteract the captopril effect observed in the captopril-alone treatment group, indicating that bradykinin degradation hasn't contributed to the effect of ACE on lung apoptosis and inflammation. Therefore, the authors concluded downstream signaling mediated by Ang II but not bradykinin in the current rat model of BPA-induced lung injury. In a parallel study, Lassila et al. [35] reported that the renin-angiotensin system, but not the kallikrein-kinin system (KKS), is an involved mechanism in cyclosporin-induced nephrotoxicity in spontaneously hypertension rat (SHR) model. Also, Al-Kuraishy et al. [36] suggested a role for the renin-angiotensin system in a rat model of gentamicin-induced nephrotoxicity. They reported a protective effect for Irbesartan, a selective angiotensin II receptor blocker, via antioxidative and anti-inflammatory mechanisms.

The ACE inhibitors and angiotensin II receptor blockers have four unique mechanisms that could explain each group's findings. First, ACE is partially responsible for the Ang II generation due to other alternative sources for Ang II generation by the noncanonical renin-independent route [37]. Ang II receptor antagonists block the Ang II effects regardless of the source of Ang II. Second, the action on ATR subtypes ATR1 and ATR2. Losartan blocks the deleterious ATR1 only; meanwhile, the seemingly protective ATR2 is free. ATR2 seems to mediate opposing effects to ATR1 [38]. Thus, losartan treatment had a superior effect in restoring the inflammatory and apoptotic markers than captopril. Third, the ACE inhibitor captopril decreases the breaking down (so increases the accumulation) of the pro-inflammatory bradykinin (BK); meanwhile, losartan lacks this effect. However, icatibant (BK B2 receptor blocker) didn't counteract the captopril effect, assuming the captopril effects were unrelated to bradykinin. Finally, Reis et al. [39] suggested JNK/ERK1/2 phosphorylation in vitro and intracellular signaling pathway activation by captopril, in addition to its suppressive capability on the ACE enzymatic activity. This might facilitate the beneficial effects of ACE inhibitors and work more effectively in different disease models.

## 5 Conclusion

BPA exposure caused lung toxicity in rats facilitated by ACE2/ACE system imbalance, leading to angiotensin II generation. Both captopril and losartan improved the lung histoarchitecture and decreased the lung injury score, but losartan was more excellent than captopril in regulating the BALF inflammatory markers, ACE, ACE2, caspase-3 gene/protein expressions, and maintaining ACE2/ACE balance. Also, the study reported RAS/angiotensin II, rather than KKS/bradykinin, as an involved mechanism in the current rat model of BPA-induced lung damage. The study shed light on the local lung RAS and its role in BPA-induced lung injury, raising attention to the potential association of BPA exposure with COVID-19 infection susceptibility by modulating the ACE2 expression, the critical mediator of SARS-CoV-2 infection. In addition, the study identified ACE2/ACE rebalance as a promising therapeutic strategy in chemical-induced lung injury.

## 6 Strengths and limitations of the study

In the authors’ opinion, one point of strength may be the used model of eight-week chronic low-dose bisphenol-A exposure which may be more relevant to the actual human exposure and to study the long-term toxicity. On the other hand, the study had few limitations. The osmotic mini pump for continuous and controlled icatibant delivery would be more reliable for dosing. The orogastric gastric route for captopril and losartan administration would be accurate for precise calculation of the given dose per kilogram body weight. The plasma levels of angiotensin peptide concentrations were not assessed, which would further emphasize the BALF and tissue levels. Also, it would be appropriate if the RAS enzyme activities and angiotensin metabolites were measured. In this study, angiotensin II is used as the only surrogate for ACE activity, and no measurements of other angiotensin peptides or ACE/ACE2 activities were performed. Furthermore, for future work, the individual levels of lung tissue ACE2 and ACE should be measured, and precise calculation of the ACE2/ACE ratio should be obtained and compared between the groups.

## Data availability statement

All the research data are present in the published version of the article, and further queries are on reasonable request.

## Funding

This research did not receive any specific grant from funding agencies in the public, commercial, or not-for-profit sectors.

## CRediT authorship contribution statement

**Ahmed A. Morsi:** Conceptualization, Formal analysis, Methodology, Writing – original draft, Writing – review & editing. **Ezat A. Mersal:** Formal analysis, Resources, Supervision, Writing – original draft. **Ahmed M. Abdelmoneim:** Formal analysis, Resources, Supervision, Writing – original draft. **Eman Mohamed Faruk:** Conceptualization, Formal analysis, Writing – original draft, Writing – review & editing. **Nehad Ahmed Sadek:** Conceptualization, Methodology, Formal analysis, Writing – review & editing. **Mohamed M. Sofii:** Formal analysis, Resources, Supervision, Writing – original draft. **Khalid Elfaki Ibrahim:** Data curation, Resources, Validation. **Hatem J. Aljanfawe:** Data curation, Validation. **Iman Elmadhoun:** Data curation, Validation. **Wejdan Mubarak:** Data curation, Validation. **Mashael Malik Mahmoud:** Data curation, Validation. **Mohamed S. Salim:** Data curation, Investigation, Project administration, Resources, Visualization, Writing – original draft.

## Declaration of competing interest

The authors declare that they have no known competing financial interests or personal relationships that could have appeared to influence the work reported in this paper.
